# Three-Dimensional Reconstruction of Oral Tongue Squamous Cell Carcinoma at Invasion Front

**DOI:** 10.1155/2013/482765

**Published:** 2013-10-21

**Authors:** Tomoo Kudo, Yoshihito Shimazu, Hisao Yagishita, Toshiyuki Izumo, Yuuichi Soeno, Kaori Sato, Yuji Taya, Takaaki Aoba

**Affiliations:** ^1^Department of Pathology, Hyogo College of Medicine, 1-1 Mukogawa-cho, Nishinomiya, Hyogo 663-8501, Japan; ^2^Department of Pathology, School of Life Dentistry at Tokyo, The Nippon Dental University, 1-9-20 Fujimi, Chiyoda-ku, Tokyo 102-8159, Japan; ^3^Division of Oral Diagnosis, Dental and Maxillofacial Radiology and Oral Pathology Diagnostic Services, The Nippon Dental University Hospital, 2-3-16 Fujimi, Chiyoda-ku, Tokyo 102-8158, Japan; ^4^Department of Diagnostic Oral Pathology, Graduate School of Medical and Dental Science, Tokyo Medical and Dental University, 1-5-45 Yushima, Bunkyo-ku, Tokyo 133-8549, Japan

## Abstract

We conducted three-dimensional (3D) reconstruction of oral tongue squamous cell carcinoma (OTSCC) using serial histological sections to visualize the architecture of invasive tumors. Fourteen OTSCC cases were collected from archival paraffin-embedded specimens. Based on a pathodiagnostic survey of whole cancer lesions, a core tissue specimen (3 mm in diameter) was dissected out from the deep invasion front using a paraffin tissue microarray. Serial sections (4 **μ**m thick) were double immunostained with pan-cytokeratin and Ki67 antibodies and digitized images were acquired using virtual microscopy. For 3D reconstruction, image registration and RGB color segmentation were automated using ImageJ software to avoid operator-dependent subjective errors. Based on the 3D tumor architecture, we classified the mode of invasion into four types: pushing and bulky architecture; trabecular architecture; diffuse spreading; and special forms. Direct visualization and quantitative assessment of the parenchymal-stromal border provide a new dimension in our understanding of OTSCC architecture. These 3D morphometric analyses also ascertained that cell invasion (individually and collectively) occurs at the deep invasive front of the OTSCC. These results demonstrate the advantages of histology-based 3D reconstruction for evaluating tumor architecture and its potential for a wide range of applications.

## 1. Introduction

Oral tongue squamous cell carcinoma (OTSCC) is the most prevalent head and neck cancer, and the presence of occult neck metastases is the main predictor of outcome in patients with early-stage OTSCC [1–4]. In the literature, it has been documented that the depth of infiltration of the primary tumor correlates significantly with the rate of regional nodal metastases [5–7], but the specific cut-off value for the depth of infiltration distinguishing high-risk and low-risk patients remains controversial [8, 9]. Most recently, Ganly et al. [10] indicated that patients with pathologic T1-T2/N0 OTSCC had a greater than expected rate of neck failure and that failure occurred predominantly in patients with primary tumors greater than 4 mm in thickness.

It is well known that the histological features of OTSCC differ widely between different regions within the same tumor, and much effort has been given to explore specific invasion patterns that may have prognostic value. A consensus is developing that different patterns of tumor invasion are determined by the interaction between cancer cells and the circumscribing stromal environment [11, 12] and that the most useful prognostic information can be deduced from the deepest invasive front of the tumor, where the most aggressive cells are presumably found [13–15]. In the literature, the deep invasion patterns of OTSCC are usually classified into three or four categories, such as (i) pushing types with well-delineated infiltrating borders; (ii) diffuse types with infiltrating solid cords, bands, and/or strands; and (iii) destructive types with widely spread cellular dissociation in small groups and/or single cells [8, 16–18]. With respect to the relationship between the invasion pattern and clinical outcome, many studies conclude that the prognosis of patients whose OTSCC exhibits a pushing-border invasion pattern is better than for those with diffuse and destructive invasion types [8, 14–18]. Nevertheless, the propensity of an OTSCC to metastasize subclinically remains hard to predict solely through microscopic examination [19, 20]. 

An important consideration is that histopathological features of tumor malignancy are usually diagnosed from two-dimensional (2D) microscopic findings, whereas tumor progression actually occurs within a three dimensional (3D) microenvironment. Recent developments in computing power and software sophistication have facilitated the 3D reconstruction of anatomical and pathological structures [21–24]. We previously described the development of technology to reconstruct the tumor-host environment using serial histological sections of archival paraffin-embedded human cancer specimens [25]. In the current paper, we use double immunostaining with pan-cytokeratin and Ki67 antibodies to correlate microscopic 2D and 3D findings with respect to heterogeneous tumor architecture in T1/T2 OTSCC at the deepest invasion front. The main objective of the present study was to visualize directly the tissue architecture at the tumor-host interface and to assess quantitatively the mode of invasion for single cells and cell clumps within the 3D microenvironment.

## 2. Materials and Methods

### 2.1. Patients and Tongue Cancer Cases

For the present study, we included 14 patients with early-stage (cT1-T2/N0) OTSCC who were treated at Nippon Dental University and Saitama Cancer Institute between 2000 and 2009. Patient records were retrieved from the archival OTSCC records based on the following criteria: (a) patients had radical surgery as their first line of management (i.e., without preoperative chemotherapy and radiotherapy); (b) paraffin-embedded tissue specimens of the primary tumor were available; and (c) clinical follow-up data for a minimum of 24 months or until death were recorded. The patient group included nine men and five women with a mean age of 58 years (range: 33–82 years) at the time of diagnosis. Although all patients were N0 at presentation, eight had occult neck lesions discovered during the follow-up without failure at the primary site. Based on microscopic images of whole tumor lesions (see Supplementary Material Plate S1 available online at http://dx.doi.org/10.1155/2013/482765), tumor growth patterns were classified into three types, that is, superficial spreading, exophytic, and endophytic. The clinical and pathological features of these OTSCC cases are shown in Table 1. The use of archival human tissues in this study was in accordance with the provisions of the Declaration of Helsinki for research involving human tissue and was approved by the Institutional Review Boards of Nippon Dental University and Saitama Cancer Institute.

### 2.2. Preparation of Histological Sections

**Figure 1:**
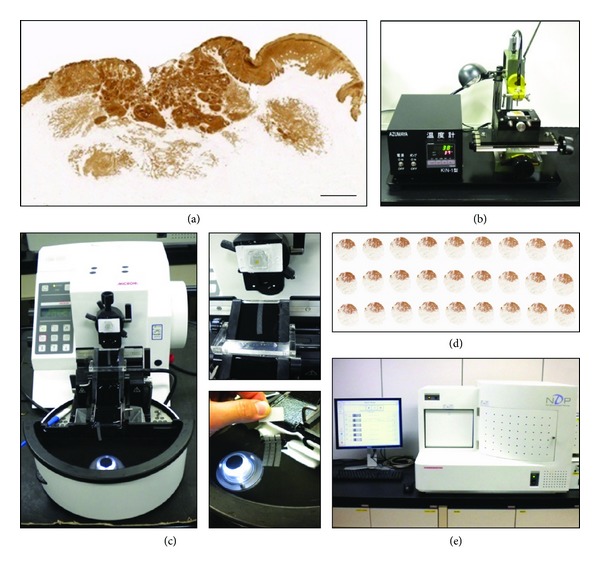
(a) Original pan-cytokeratin-stained image of an OTSCC lesion. Bar = 1 mm. (b) Tissue-array apparatus used to collect a core biopsy of 3 mm in diameter from the deepest invasion front (see Plate S1). (c) Preparation of serial sections (4 *μ*m thick) from the core specimen using a rotary microtome. The serial sections were conveyed *via* continuous laminar water flow directly from the knife edge to the heated water bath. (d) Three rows of 9 sections were mounted on each glass slide for immunostaining with Ki67 and CK cocktail. (e) All histological images were digitized using virtual microscopy.

We first reexamined primary tumor lesions from the 14 patients by preparing both HE-stained and cytokeratin-immunostained sections from the archival formalin-fixed, paraffin-embedded tissue blocks. The depth of invasion was measured from the level of the nearest adjacent normal mucosa to the extent of the deepest tumor invasion into the tongue musculature. Taking into account the observed locoregional heterogeneous histological features, we collected core specimens of 3 mm in diameter from multiple deep invasion sites of the whole tumor lesion using tissue-array apparatus (Model KIM-I, Azumaya, Tokyo, Japan; Figure 1(b)). Three-dimensional reconstruction and morphometric data are presented for a single representative site for each SCC case (Supplementary Material, Plate S1). The selected tissue core specimen was reembedded in paraffin and consecutive histological sections of 4 *μ*m thickness were prepared using an electronic motorized rotary microtome (Microm HM 355S, MICROM International GmbH, Walldorf, Germany; Figure 1(c)), which has been used successfully by others to prepare serial sections [22]. One crucial technical point is the use of a tissue core specimen of 3 mm in diameter, which enabled us to consistently obtain from the region of interest 80–120 serial sections of equal thickness and with minimal distortion (Figure 1(c)). These serial sections were mounted on glass slides, usually in three rows of 8-9 sections (Figure 1(d)). Because it minimizes the number of glass slides used for immunostaining and, as described later, facilitates automated image registration, this regimented arrangement of circular serial sections on a glass slide aided the efficient execution of our histology-based 3D reconstructions, which would otherwise be rather time and labor intensive.

### 2.3. Immunohistochemistry

We first established the immunoreactivity profiles of OTSCC lesions using a range of anticytokeratin antibodies, because cancer cells are usually heterogeneous with respect to their cytokeratin expression. From this profiling, we selected a cocktail of three antibodies (AE1/AE3, 34*β*E12, and MNF116; hereafter called CK) for our 3D reconstruction, and this CK cocktail highlighted infiltrating cancer cells and clumps at the invasion front as described in the literature [20]. Before immunostaining the serial sections, slides were deparaffinized in xylene for 3 × 5 min, rehydrated through graded ethanol to distilled water, incubated for 20 min with 3% hydrogen peroxidase in PBS to inhibit endogenous peroxidase activity, and then heated in TE buffer (0.01 M Tris-HCl plus 1 mM EDTA; pH9.0) for 60 min at 98°C for antigen retrieval. After cooling to room temperature (~60 min), the sections were incubated in 5% skimmed milk in PBS for 20 min at room temperature to block nonspecific protein binding. For double immunostaining, the sections were first incubated overnight at 4°C with Ki67 primary antibody (MIB-1, 1 : 100; DAKO, Tokyo, Japan) and then developed using Vectastain Elite ABC kits (mouse; Vector Laboratories Inc., Burlingame, CA, USA). Vector SG (SK-4700, Blue/Gray; Vector Laboratories Inc.) was used as the substrate for the peroxidase-mediated reaction. After Ki67 immunostaining, the sections were reheated in TE buffer in a microwave oven for 5 min at 90°C for antigen retrieval. For detection of tumor parenchyma using the CK cocktail, the same sections were processed using a labeled streptavidin-biotin (LSAB) method according to the Ventana DAB Universal Kit and an automated staining apparatus (NexES IHC, Ventana Medical Systems, Oro Valley, AZ, USA). Slides were mounted with EntellantNew (MERCK, Darmstad, Germany).

### 2.4. Histological Image Digitization

Red-green-blue (RGB) color images of consecutive histological sections were acquired using a 20x objective with a virtual microscope (NanoZoomer HT, Hamamatsu Photonics, Hamamatsu, Japan) (Figure 1(e)). To ensure reproducible image acquisition, color balance correction was conducted using a blank reference slide standard according to the manufacturer's procedure. The manufacturer also provided viewing software for which the scanning mode can be freely adjusted to automatically capture individual circular histological areas on a glass slide according to their numerical order. After storage of an entire series of histological digitized images (resolution of 0.92 *μ*m per pixel, 5120 × 4096 pixels, 60 MB, and 32-bit RGB TIFF format), the entire circular tumor area was transposed onto a desktop computer (see below) using frame grabber software (TRI-SRF2, Ratoc System Engineering Ltd, Tokyo, Japan).

### 2.5. Image Registration, Segmentation of Tissue Components, and Geometry Reconstruction

Image registration (rough and fine alignment of consecutive histological images) and segmentation (identification of the boundaries of target structures in images) were conducted using open-source ImageJ software that is recognized as providing practical solutions for managing memory and automated approaches in 3D registration and visualization [26]. Specifically, we used ImageJ plugins (StackReg, TurboReg, and Color Deconvolution) for computation of image registration and RGB color segmentation. This open-source software has the great advantage of providing color-segmentation channels specific for the DAB and Vector SG used in our double-immunostained sections.

**Figure 2:**
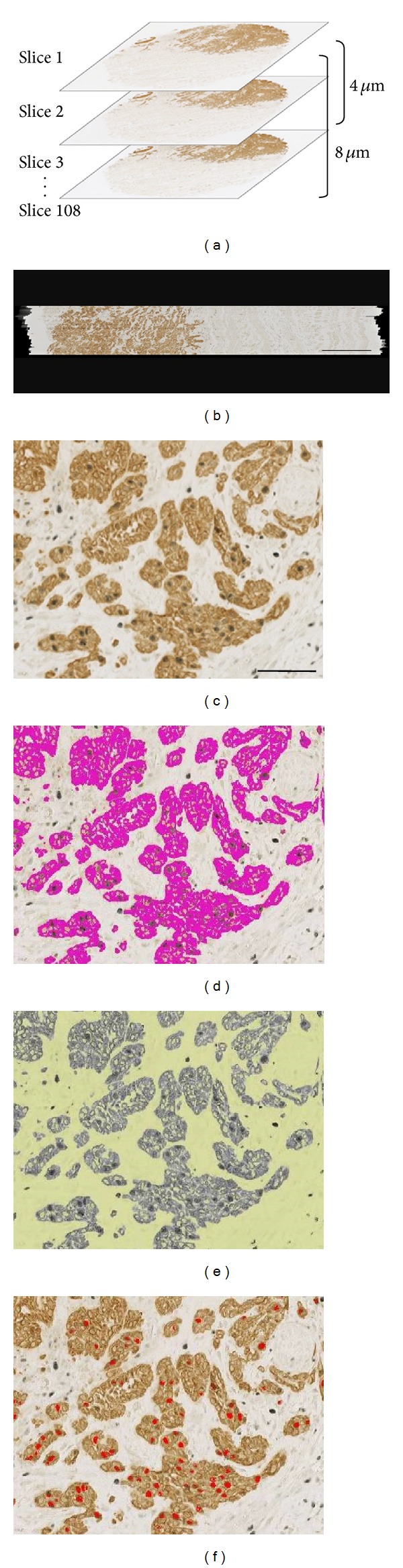
(a) Image registration of consecutive histological images in the *z*-axis. (b) Sagittal cross-cut view through the center of an image stack comprising 108 serial sections, showing successful superimposition without marked irregularity or distortion in interior tissue geometry. Bar = 0.5 mm.((c)–(e)) Segmentation of cancer cells and Ki67-positive nuclei: (c) original double-immunostained image with DAB and Vector SG as chromogens; (d) segmentation of CK-positive cancer cell cytoplasm, shown as purple pseudocolor (note that Ki67-negative nuclei remained open within the segmented cytoplasm); and (e) stromal region (yellow) after subtraction of the tumor parenchyma (see text for details); (f) Ki67-positive nuclei (red) attained by ImageJ SG color segmentation. Bar = 50 *μ*m.

In this study, superimposition of 80–108 consecutively captured images (Figure 2(a)) was accomplished successfully in an automated, algorithm-driven manner. By browsing through an image stack, it was verified that the tumor architecture was smoothly continuous between adjacent slices with no marked irregularity or distortion in interior tumor geometry and surface contour delineation (Figure 2(b)). One crucial condition for accomplishing the automated image registration is the preparation of regularly-oriented good-quality serial sections, since computation for alignment of serial sections depends on the performance of an approximate initial registration to correct for shifts and rotations of sections on the glass slides. It is also notable that we used no algorithmic image transformation, such as stretching or shrinking images in the *x*-*y* dimension to adjust for small misalignments between sections; in our experience of image registration, the introduction of the ImageJ affine image transformer actually results in deterioration of the quality of the image stack rather than improvement.

Image segmentation of tumor architecture (Figures 2(c)–2(e)) was performed using ImageJ DAB-color segmentation based on CK-positive immunohistochemical features of SCC cells. Following the segmentation of the tumor parenchyma volume, the RGB color-based volume data were inverted in a form of binary code. Thereafter, the stroma volume was calculated by subtracting the parenchyma volume from the whole tissue volume. According to the same computation logics, all Ki67-positive nuclei were labeled according to the ImageJ SG-color segmentation procedure (Figure 2(f)) and then segmented into two fractions: nuclei embedded within the CK-positive cancer cytoplasm and nuclei within the whole stromal volume. During this segmentation of nuclei, we adopted the criterion that all objects assigned as nuclei must be connected between at least two consecutive images. Thus, any binary signals in a single image plane with no connection to any corresponding objects in adjacent images were deleted as noise. The results of segmentation for each microscopic field were validated by overlaying the segmented elements on the original immunostained images on a computer screen.

Finally, a serial stack of images was used to reconstruct the 3D configuration of the specimen. *Z*-depth was adjusted to the thickness of each slice (4 *μ*m) to give an accurate 3D representation of tissue volume. Three-dimensional volume rendering of the architecture of the whole tumor and of segmented components was performed using VG Studio Max software (Volume Graphics Ltd, Heidelberg, Germany), which improves the quality of 3D visualization by adjusting the opacity and color of target components in the reconstructed space.

### 2.6. Three-Dimensional Morphometry of Tumor Architecture and Proliferation Activity

Based on the 3D volumetric information obtained, quantitative assessment was conducted using RATOC SRF2 software. The parameters of interest were the volumes of the tumor parenchyma and stroma (in mm^3^); the area of the segmented parenchymal-stromal border (in mm^2^); and the numbers of Ki67-positive nuclei and infiltrating cancer cells or clumps. To measure cancer infiltration, we labeled and counted only cancer cells or clumps that had detached from each other and were completely surrounded by stroma. Any tumor components in contact with the outer surfaces of an image stack (i.e., both top and bottom image planes and lateral circularly-cut margins) were deleted from the pool of the putative infiltrating cancer cells or clumps. We also conducted a computational test with voxel-based dilation/shrinkage functions of the labeled components to validate the continuity of tumor architecture or detachment of cancer cells from neighboring cancer foci in the 3D space.

### 2.7. Computer Hardware

The computer system used to run the 3D reconstruction and visualization consisted of an Intel Xeon computer with a 3.0 GHz processor, 32 GB RAM, Windows XP professional 64-bit edition, a graphics video card NVIDIA Quadro FX 5600 with 1.5 GB RAM, a 24-inch dual monitor system, and 500 GB internal disk drive. This computer was equipped with all necessary software as described above.

## 3. Results

### 3.1. Three-Dimensional Visualization of Tumor Parenchymal-Stromal Border at the Invasion Front

**Figure 3:**
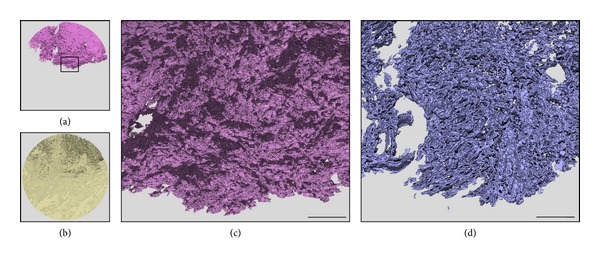
(a, b) 3D view of the segmented tumor parenchymal (purple) and stromal (yellow) regions, respectively. (c) Enlargement of the CK-positive tumor architecture in the rectangle shown in (a), showing in detail the interconnected tumor texture and channels or holes corresponding to penetrating stromal strands. (d) Labyrinthine architecture of the tumor parenchymal-stromal border, which was virtually displayed in a plane of one voxel in width. Note that this 3D image was constructed using 30 serial sections to improve the visibility of the tortuous architecture. Bar = 0.5 mm.

Figure 3 shows an example of the segregation of the CK-positive tumor parenchyma (Figure 3(a)) and the adjacent stromal space (Figure 3(b)). In this OTSCC case (case C in Table 2), the segmented tumor mass appears bulky at low magnification, but a magnified 3D visualization reveals that the parenchymal-stromal border has a rough surface texture where small tumor cords and strands are connected to each other with narrow stromal penetration (Figure 3(c)). When the segmented parenchymal-stromal border was delineated in a virtual space as a plane of one voxel in width, the labyrinthine structure extending into the massive intratumor space can be fully appreciated (Figure 3(d)). As shown in Table 2, based on 3D morphometry, this bulky tumor architecture and its intricate tumor-host border correspond numerically to a parenchymal volume of 2.95 mm^3^, a border area of 61.4 mm^2^, and a surface area per volume of 97.5 mm^−1^.

### 3.2. Three-Dimensional Tumor Architecture at the Invasion Front

**Figure 4:**
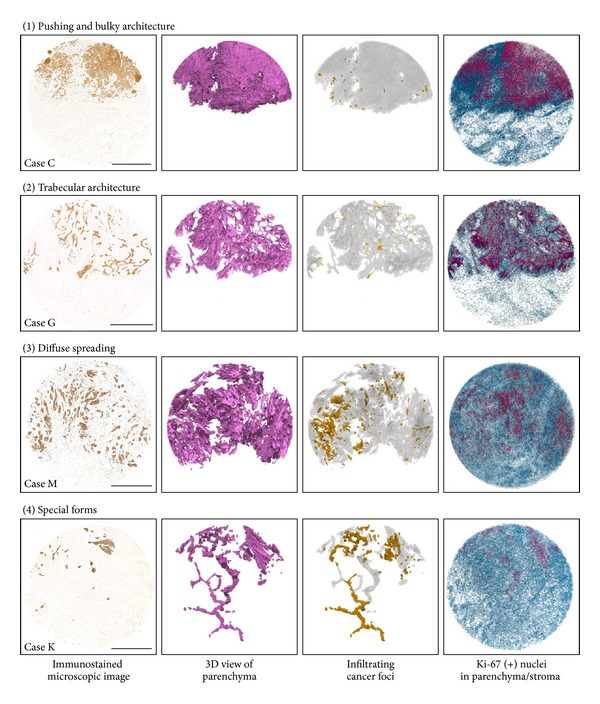
Four types of tumor architecture and mitotic activity at the OTSCC invasion front. Images are from left to right: the immunostained microscopic (2D) image; 3D view of the segmented tumor parenchyma; 3D view of infiltrating cancer foci (yellow) detached in all dimensions from the bulk tumor parenchyma (gray); and Ki67-positive nuclei in the tumor parenchyma (red) and stroma (blue). Additional 3D data obtained from the remaining 10 OTSCC cases can be found in Supplementary Material Plates S2 and S3. Bar = 1 mm.

Figure 4 compares microscopic (2D) and reconstructed (3D) images of tumor architecture and invasion mode in four cases of OTSCC; the remaining data from the other OTSCC cases are presented in Supplementary Material Plates S2 and S3. Using the CK cocktail to label cancer cells, 2D views reveal heterogeneity in the patterns of cancer cell invasion both between OTSCC cases and within individual cases. Among the infiltrating cancer cells observed on 2D images, the 3D segmentation protocol enabled the visualization of discrete cancer cells and clumps that were detached from each other and completely encased in the stroma. We herein use the term “discohesive cancer foci” to designate a pool of infiltrating cancer cells segmented in 3D space. We also addressed locoregional differences in mitotic activity as indicated by the density of Ki67-positive nuclei in the parenchymal and stromal segments.Based on the 3D features of tumor architecture, we discerned four types: (1) pushing and bulky architecture with short finger-like projections; (2) trabecular architecture with strands and cords; (3) diffuse spreading; and (4) special forms.

1. *Pushing and bulky architecture with short finger-like projections*: this type of SCC case was typified by one or a few solid tumor masses with delineated pushing infiltration at the advancing front. CK-immunostaining disclosed a fine network of tumor strands and cords as well as massive tumor growth on the 2D image. However, when viewed in 3D, we discovered that the majority of tumor strands and cords were topologically connected, giving rise to a massive tumor volume with narrow stromal penetration, as depicted in Figure 3. This 3D segmentation also verified the presence and localized the distribution of discohesive cancer foci along the tumor-host border. Two common trends were noted in the mitotic activities of the parenchyma and stroma: (a) the tumor periphery in contact with the region of stromal penetration usually showed higher mitotic activity than the compact core of the tumor mass and (b) dense Ki67 nuclear staining in the narrow stromal margins adjacent to the pushing tumor mass, most of which was attributable to infiltrating leukocytes and proliferating endothelial cells (data not included).
2. *Trabecular architecture with strands and cords*: in 2D microscopic images acquired from this type of SCC case, cancer cells were infiltrating diffusely in various forms of extended tortuous cords and strands, which in part gave rise to an interconnected alveolar appearance. The 3D reconstruction verified the continuity of most cords and strands, which generated a honeycomb architecture that permeated through an extensive stromal volume. Discohesive cancer foci were also localized along the trabecular tumor mass, usually with no evidence of distant migration into the host stromal environment. The Ki67-positive nuclei were distributed randomly and homogeneously throughout the tumor trabeculae reflecting the close spatial contract between the cancer cells and their adjacent stromal environment.
3. *Diffuse spreading*: on the 2D images, this type of tumor invasion was characterized by a scattering of small cancer foci or cells in the muscular structure of the tongue, a finding substantiated by 3D analysis, which verified the presence of many discohesive cancer foci consisting of single or a few cancer cells, as shown below. It is notable that mitotic activity was not prominent in these infiltrating cancer cells, but was extended widely into the host environment rather than being localized at the tumor margin.
4. *Special forms*: on the 2D images, this type of tumor invasion featured localized infiltration of only a small number of cancer cells or clumps into the deep tongue musculature. The 3D segmentation confirmed that this punctate appearance corresponded to a cross-sectional view of slender, tortuous strands, and cords extending into the intermuscular space without massive destruction of the muscular structure of the tongue. In accordance with their limited destructive capacity, the distribution of Ki67-positive nuclei through the tumor architecture was sparse and heterogeneous in the tortuous strands and cords.

### 3.3. Morphometric Features of 3D Tumor Architecture at the Invasion Front

Table 2 gives the results of 3D morphometric analyses of 14 OTSCC cases studied. These OTSCC cases, labeled A–N, are listed in order of the measured depth of infiltration. By initially dividing these cases into three classes (shallow, <3 mm infiltration; intermediate, 3–5 mm; and deep, >5 mm), we found that four out of the five OTSCC cases with “pushing and bulky architecture” belonged to the shallow class (the other being in the intermediate class), while three out of four cases with “trabecular architecture” were in the intermediate class (the other being in the shallow class). All of the “diffuse spreading” and “special forms” cases belonged to the deep class, except for one case that featured diffuse spreading but only shallow infiltration.

Quantitative analyses showed several unique features of OTSCC cases depending on their 3D architecture. The reconstructed tissue volume ranged from 2.19 to 2.95 mm^3^, depending on the number of serial sections used. The volume of segmented tumor parenchyma varied widely from 1 to 42% of total tissue volume; as expected, the volume values were generally higher in the “pushing and bulky architecture” group and the lowest in the “special forms” group. In accordance with the intricate tumor-host border as depicted in Figure 3, the values of border area also varied widely, from 3.8 to 81.8 mm^2^. By normalizing the border area (*S*) against the tumor parenchyma volume (*V*_p_), it is notable that five OTSCC cases in the “pushing and bulky architecture” group yielded relatively uniform values (average *S*/*V*_p_ = 78.6 ± 18.2 mm^−1^), considerably lower than the average *S*/*V*_p_ values for four cases of “trabecular architecture” (81.6 ± 45.5 mm^−1^) and three cases of “diffuse spreading” (136.4 ± 40.6 mm^−1^). The spatial resolution of the 3D reconstruction was sufficient to determine 10^3^–10^5^ Ki67-positive nuclei in the tumor parenchyma of each sample. By normalizing the number of Ki67-positive nuclei against tumor parenchyma volume, we revealed 6-fold differences in the average mitotic activity between OTSCC cases, although there were no coherent architecture-dependent trends. Table 2 also shows the number of discohesive cancer foci. These varied from 2 to 232 in number and, when normalized as a percentage of the tumor parenchyma volume, it was ascertained that the contribution of detached cancer cells to the total tumor mass was only marginal in the “pushing and bulky architecture” group but was markedly increased in the “diffuse spreading” and “special forms” groups.

### 3.4. Size Distribution of Discohesive Cancer Foci Segmented at the Advancing Front

**Figure 5:**
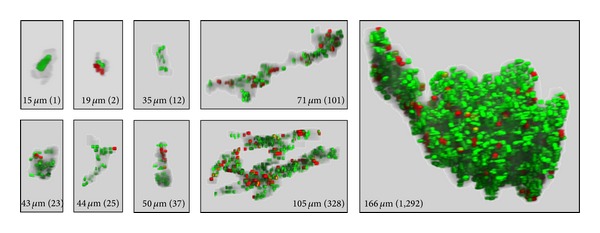
Three-dimensional view of individual cancer foci segmented at the invasion front. The size of the corresponding cancer mass is expressed in terms of diameter of a sphere having the same volume. The number in parentheses indicates the number of nuclei segmented from the cancer volume. Red: Ki67-positive nuclei; green: Ki67-negative nuclei (see text for nuclear segmentation).

Table 3 shows the size distribution analysis of 966 discohesive cancer foci segmented at the invasive front of 14 OTSCC cases. In this analysis, the volume of each segmented cancer mass was determined in the reconstructed 3D space and, for the sake of simplicity, the size distribution was expressed in terms of the diameter of a sphere of the same volume. Notably, the majority of discohesive cancer foci were small in size, with the greatest population (39%) having a diameter ranging between 16 to 20 *μ*m. To gain further insight into the cellular constitution of these discohesive cancer foci, we developed a computational algorithm using Ratoc TRI-SRF2 software to distinguish Ki67-positive and Ki67-negative nuclei in individual cancer foci. Ki67-negative nuclei were designated as open space circumscribed by CK-positive cytoplasm (see Figure 2(d)); noise images were deleted in the same way as applied for segmentation of Ki67-positive nuclei. To date, we have completed 3D analyses and visualization of 50 discohesive cancer foci randomly selected from different size ranges and OTSCC cases.  Figure 5 shows representative 3D images of Ki67-positive (red) and Ki67-negative (green) nuclei in discohesive cancer foci. The smallest consisted of a single CK-positive cell with a Ki67-negative nucleus, while the largest analyzed so far included a total of 1,292 nuclei, comprising 277 Ki67-positive and 1,015 Ki67-negative nuclei. It is important to note that the majority (68%) of discohesive cancer foci with diameters <25 *μ*m contained only a few cancer cells. In addition to highlighting the differences in the number of nuclei, 3D visualization of individual discohesive cancer foci disclosed their heterogeneous morphological features, for example, spheroidal, amoeboid, branching or stretching with extension of projections into the surrounding environment.

## 4. Discussion

The invasion mode of OTSCC is usually assessed in 2D microscopic images. However, the actual 3D configuration of tumor invasion cannot be extrapolated on the basis of 2D examination alone. For instance, an anastomosing network of tumor strands or cords within the tissue volume may appear in 2D sections as punctate cancer cells or islands. In the present study, this type of misleading appearance was most obvious in the OTSCC cases classified as “special forms”. Three-dimensional reconstruction using double immunostaining with CK and Ki67 antibodies is useful in segregating the tumor parenchyma from the surrounding stroma and to assess locoregional heterogeneity in the mitotic activity of cancer cells. The direct visualization and quantitative assessment of the tumor-host border, which cannot be extrapolated from 2D examination of histological sections, provide a new dimension in our understanding of OTSCC architecture.

In the literature, the mode or mechanism of tumor invasion and metastasis has been classified into two categories: cancer cells can disseminate as individual cells (referred to as “individual cell migration”) or in the form of solid cell strands, sheets, or clusters (known as “collective migration”) [27]. From a practical point of view, pathologists use “tumor budding” as a prognostic factor in various human cancers [28–34]. Tumor budding is usually defined as small cell clusters composed of less than five cells at the invasive tumor margin [28, 31]. According to Bryne's malignancy grading system of oral cancer, invasive tumor islands were subdivided into two classes: >15 cells and <15 cells per island [17]. In the present study, we identified 966 discohesive cancer foci at the invasive front of 14 OTSCC cases. At present, our 3D data remain limited with respect to the morphology and cellular constitution of these foci because segmentation and morphometry of individual foci are time-consuming and labor-intensive tasks. However, the results obtained so far support the theory that both individual-cell and collective migration processes occur at the OTSCC invasion front and that the majority of discohesive cancer foci comprise only a few cancer cells.

In relation to the mechanism of detachment of carcinoma cells from the primary tumor mass, it is widely accepted that these alter their phenotypes with the progression of malignancy, resulting in a loss of cell attachment and/or an epithelial-mesenchymal transition (EMT) [35, 36]. Regarding the possibility of EMT in OTSCC at the invasion front, it should be noted that our RGB color segmentation depended on the immunoreactivity of cancer cells for a cocktail of anti-CK antibodies, so that segmented carcinoma cells undergoing single-cell invasion retain their epithelial phenotype without experiencing a complete EMT. Obviously, it is important to characterize the phenotypic changes that accompany the initiation and progression of discohesive invasion of SCC cells, and this future work could investigate additional putative immunolabels such as E-cadherin, vimentin, and Snail.

As described in the Introduction, the clinico-pathological predictors of outcome in patients with early stage OTSCC have been intensely studied. The critical depth of infiltration of the primary tumor in connection with nodal metastasis has been estimated to be around 4 mm [5, 9, 10]. In the present study, we examined 14 cases of OTSCC showing a range of infiltration depths from 1.9 to 10.6 mm. We first investigated whether the depth of infiltration significantly predicted occult neck metastases but rejected this hypothesis on the basis that two patients with shallow infiltration (2.4 and 2.5 mm) had occult metastasis. Next, we critically evaluated the relationship between the frequency of discohesive cancer foci and the depth of infiltration. Table 3 shows that the frequency and size of discohesive cancer foci increase with the depth of infiltration; this trend was most prominent in the deep-infiltrating OTSCC cases with diffuse spreading and special forms. Of more importance to pathological diagnosis, however, are the findings that detachment of a single or a few cancer cells occurred in the shallow infiltration groups regardless of the type of tumor architecture and that occult neck metastasis was associated with all types of 3D tumor architecture and invasion modes, as exemplified in case B, which had a delineated pushing border and only two discohesive cancer foci in the analyzed 3D space. Although the mechanisms underlying the capacity of shallow-infiltrating OTSCCs to metastasize via lymphatic vessels have not yet been fully elucidated, it is pertinent to point out that even a massive OTSCC with a pushing border possessed a wide surface area in contact with the stromal environment, including blood/lymph vessels. Ohno et al. [37] previously reported that OTSCC cells in clumps contact pre-existing dilated lymphatic vessels and break through the thin-walled lymphatic vessel to enter the lumen. In our seminal 3D reconstruction in combination with immunolabels of tumor and vascular endothelial cells, we also observed that the tips of the primary tumor mass of an OTSCC frequently penetrate through thin-walled lymphatic vessels to enter the lumen, resulting in tumor emboli in the lumen. Taken together, we consider that dissemination of cancer cells via the lymphatic vessels may occur at very early stages of OTSCC development without the manifestation of metastatic growth in new environments.

## 5. Conclusion

The development of new technologies and methods is continually increasing the speed and utility of histology-based 3D reconstruction for investigating various anatomical and pathological objects [24, 26]. The present 3D image reconstruction proved the feasibility of volumetric isolation of OTSCC architecture segmented from the surrounding stroma at high spatial resolution. There is still a paucity of information regarding the mechanism of dissemination of OTSCC leading to regional lymph node and distant metastases. It will be necessary to visualize the spatial proximity of the tumor architecture and the vasculature and lymphatics in the tumor-host environment, an investigation currently ongoing in our laboratory.

## Supplementary Material

Supplementary Material Plate S1: Microscopic images of pan-cytokeratin immunostained OTSCC lesions. A tissue core specimen of 3 mm in diameter was collected from the deep invasion front (indicated by the circle). The value indicates the depth of infiltration, except for case N where measurement was not applicable. Bar = 1 mm.

## Figures and Tables

**Table 1:** Clinicopathological features of 14 patients with OTSCC.

OTSCC cases	Age (yrs)	Sex	p-T	Growth pattern^(1)^	Depth of invasion^(2)^ (mm)	Occult metastasis
A	51	M	T1	SS	1.9	−
B	59	M	T1	SS	2.4	+
C	82	F	T2	EN	2.5	+
D	52	M	T2	SS	2.6	−
E	53	M	T1	EX	2.8	−
F	52	M	T2	EX	2.9	+
G	60	M	T1	SS	3.3	−
H	78	M	T2	EN	3.5	+
I	55	F	T1	EX	3.6	−
J	57	M	T1	SS	4.6	+
K	40	F	T2	EN	7.0	+
L	76	F	T2	EN	7.6	+
M	69	F	T1	EN	10.6	+
N	33	M	T2	EN	N.A.	−

^(1)^Macroscopic growth patterns of OTSCC were classified into superficial spreading (SS), exophytic (EX), and endophytic (EN) types.

^(2)^The depth of invasion was measured from the level of the adjacent normal mucosal surface to the deepest portion of tumor invasion. In case N, the measurement was not applicable (N.A.).

**Table 2:** Results of 3D morphometry of 14 OTSCC cases.

OTSCC cases	Invasion mode^(1)^	Tissue volume^(2)^		Border area (*S*) mm^2^	*S*/*V*_p_^(3)^ mm^−1^	Ki67(+) nucleiin cancer cells^(4)^		Discohesivecancer foci^(5)^	
		Total mm^3^	Parenchyma (*V*_p_) mm^3^ (%)			Number	×10^4^/mm^3^	Number	% volume
A	DS	2.88	0.19 (6.6)	17.2	90.5	20,805	[10.9]	65	(2.83)
B	PB	2.86	0.96 (33.6)	48.3	50.3	221,803	[23.1]	2	(0.0010)
C	PB	2.95	0.56 (21.4)	61.4	97.5	61,896	[9.8]	30	(0.060)
D	PB	2.91	0.37 (12.7)	30.9	83.5	47,913	[12.9]	53	(0.0089)
E	TSC	2.74	0.47 (17.2)	18.3	38.9	23,702	[5.0]	19	(0.44)
F	PB	2.58	0.43 (16.7)	31.3	72.8	108,234	[25.1]	116	(0.26)
G	TSC	2.94	0.20 (6.8)	27.2	136.0	29,009	[14.5]	76	(0.59)
H	PB	2.19	0.92 (42.0)	81.8	88.9	88,303	[9.6]	19	(0.024)
I	TSC	2.91	0.49 (16.8)	24.4	49.8	80,410	[16.4]	7	(0.088)
J	TSC	2.59	0.11 (4.2)	11.2	101.8	18,435	[16.8]	36	(0.33)
K	SF	2.39	0.031 (1.3)	3.8	122.6	4,193	[13.5]	117	(27.1)
L	DS	2.73	0.11 (4.0)	18.4	167.3	35,156	[32.0]	232	(3.76)
M	DS	2.74	0.19 (6.9)	28.8	151.6	45,217	[23.8]	159	(8.34)
N	SF	2.91	0.032 (1.1)	27.2	850.0	9,751	[30.5]	35	(1.69)

^(1)^The abbreviations used are PB, pushing and bulky architecture; TSC, trabecular architecture with strands and cords; DS, diffuse spreading; and SF, special forms.

^(2)^The volume data correspond to the reconstructed total tissue volume and the segmented tumor parenchymal volume (*V*_p_). The number in parentheses indicates the volume ratio of *V*_p_ to the total tissue.

^(3)^ *S*/*V*_p_ indicates the ratio between tumor parenchyma-stroma border area (*S*) and tumor parenchyma volume (*V*_p_).

^(4)^The number in brackets indicates the number of Ki67-positive nuclei per tumor parenchyma volume (*V*_p_).

^(5)^The number in parentheses indicates the volume ratio of discohesive cancer foci to tumor parenchymal volume (*V*_p_).

**Table 3:** Size distribution of discohesive cancer foci segmented at the invasion front.

OTSCC cases^(1)^	A	B	C	D	E	F	G	H	I	J	K	L	M	N	Total
Depth of invasion (mm)	1.9	2.4	2.5	2.6	2.8	2.9	3.3	3.5	3.6	4.6	7.0	7.6	10.6	ND	
Invasion mode	DS	PB	PB	PB	TSC	PB	TSC	PB	TSC	TSC	SP	DS	DS	SP	
Occult metastasis	−	+	+	−	−	+	−	+	−	+	+	+	+	−	

Size (*μ*m)^(2)^	Numbers of discohesive cancer foci														

16>	4	0	0	12	1	13	4	2	0	2	1	17	18	1	75
16–20	32	1	0	29	12	73	33	5	0	13	15	80	63	23	379
21–25	18	1	13	8	3	21	14	7	0	10	21	54	32	5	207
26–30	5	0	12	2	1	4	10	3	1	7	23	33	19	2	122
31–35	3	0	4	1	0	1	5	1	2	2	12	22	8	1	62
36–40	0	0	1	0	1	2	2	0	0	1	11	9	3	0	30
41–45	1	0	0	0	0	1	1	1	1	0	10	6	7	1	29
46–50	1	0	0	1	1	1	7	0	3	1	21	11	8	2	57
51–200	1	0	0	0	0	0	0	0	0	0	3	0	1	0	5
200<	0	0	0	0	0	0	0	0	0	0	0	0	0	0	0

Total	65	2	30	53	19	116	76	19	7	36	117	232	159	35	966

^(1)^Among the clinicopathological features of 14 OTSCC cases, most relevant terms are relisted here (see Tables 1 and 2 for the details).

^(2)^The size of the segmented discohesive cancer foci is expressed in terms of diameter of a sphere of the same volume.
