# Supplementary material for: Three-Dimensional Reconstruction of Oral Tongue Squamous Cell Carcinoma at Invasion Front
Source: Int J Dent. 2013 Oct 21;2013:482765. doi: 10.1155/2013/482765 (PMC3818895; doi:10.1155/2013/482765)

Supplementary Material Plate S1

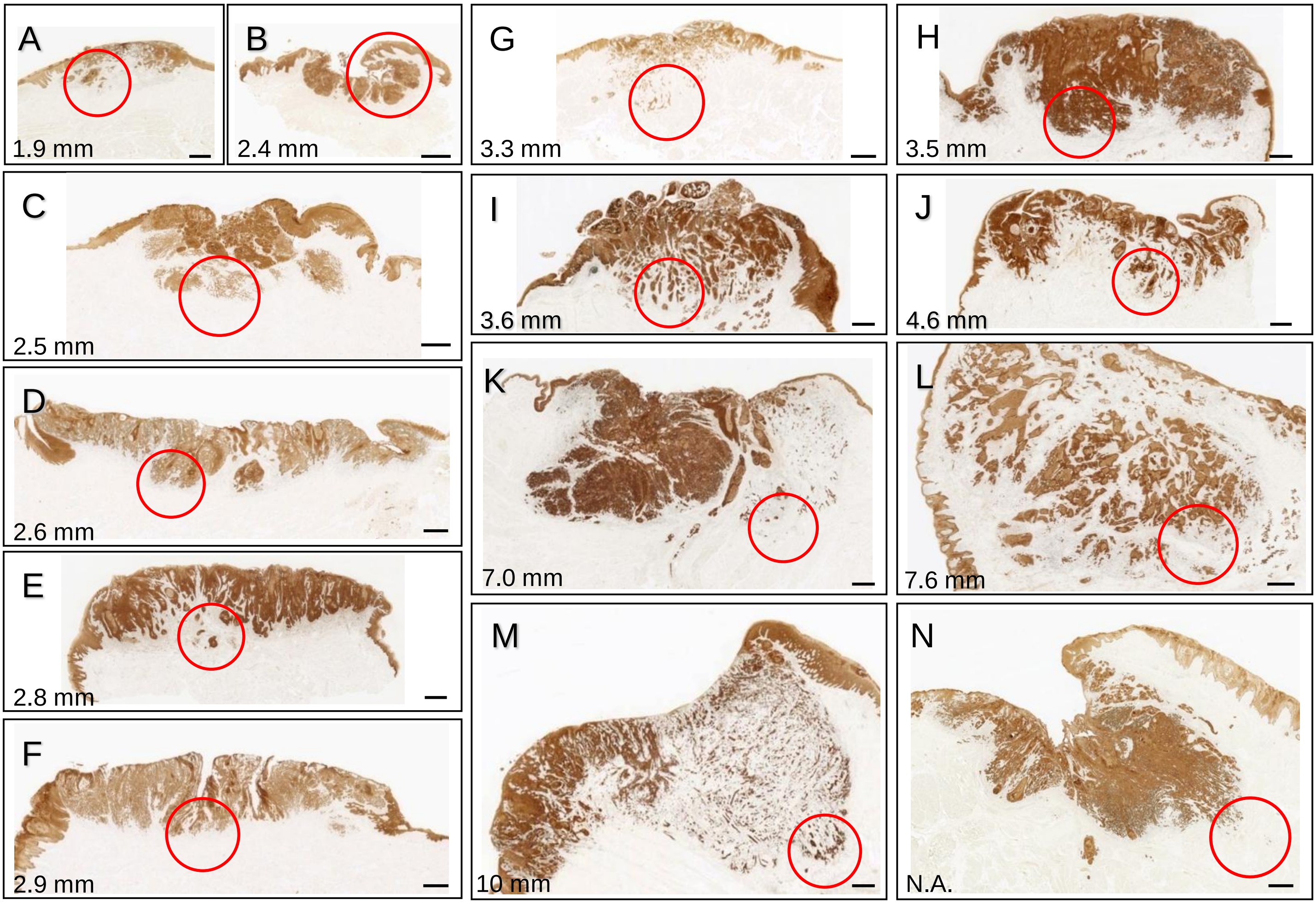

Supplementary Material Plate S2

(1) Pushing and bulky architecture

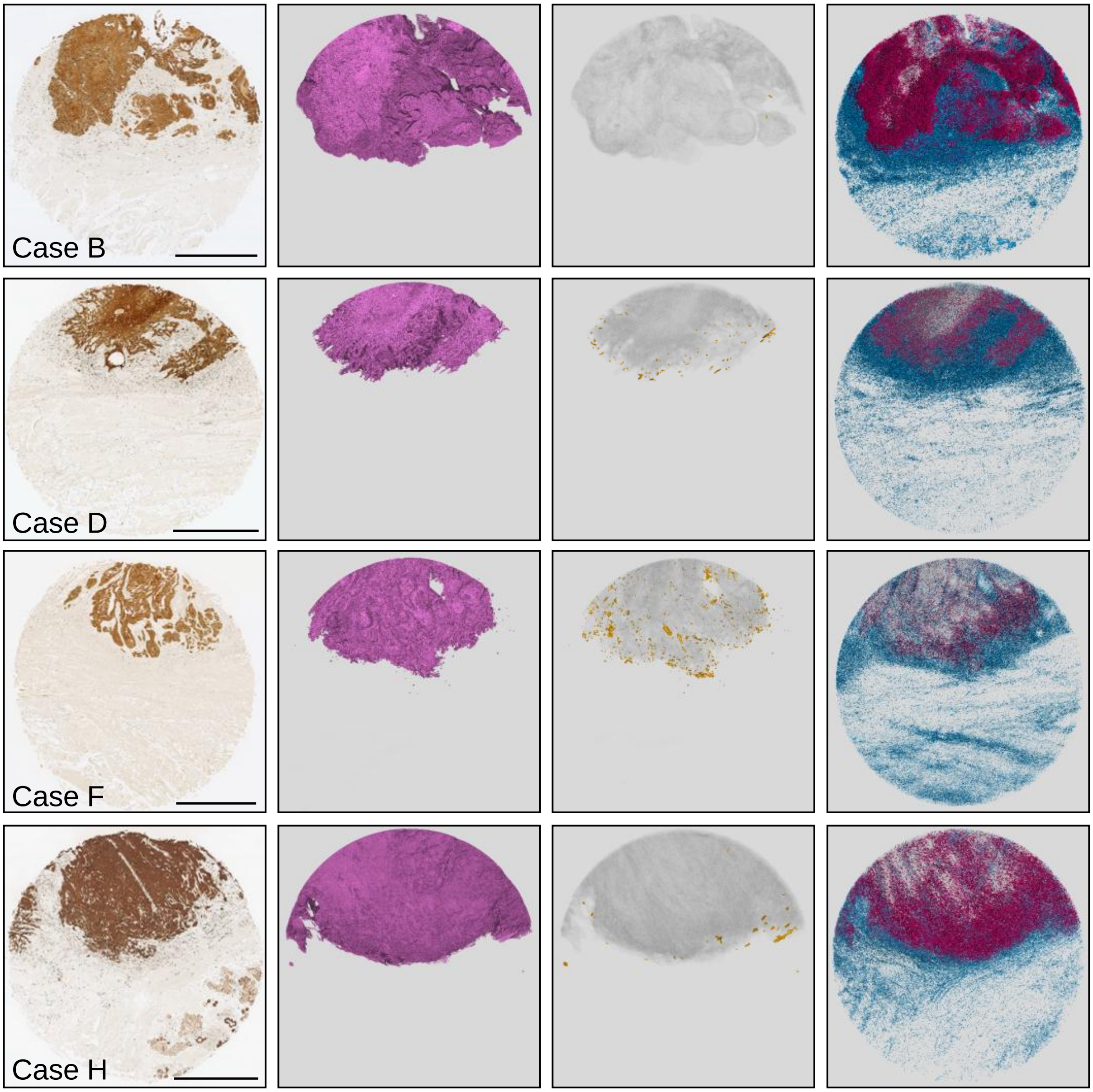

Supplementary Material Plate S3

(2) Trabecular architecture

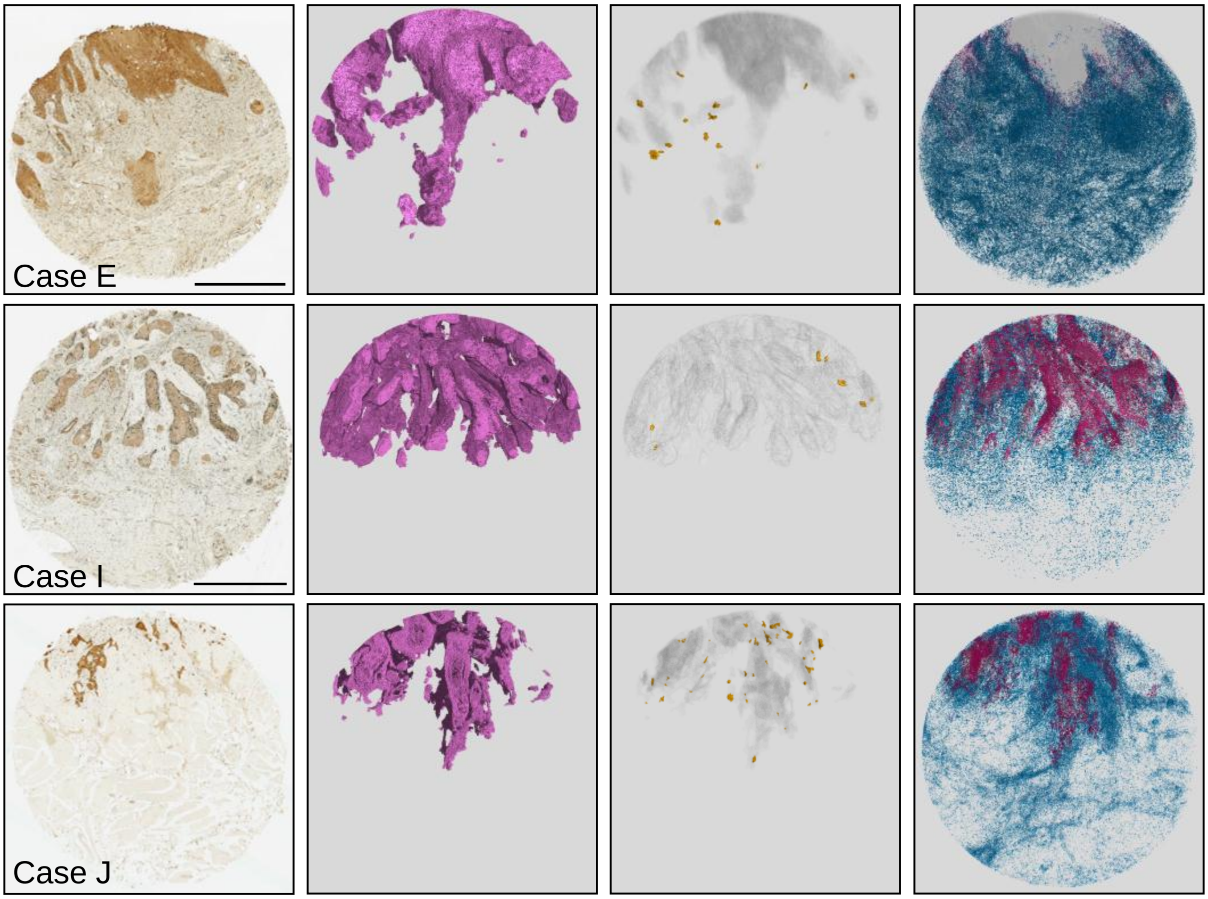

(3) Diffuse spreading

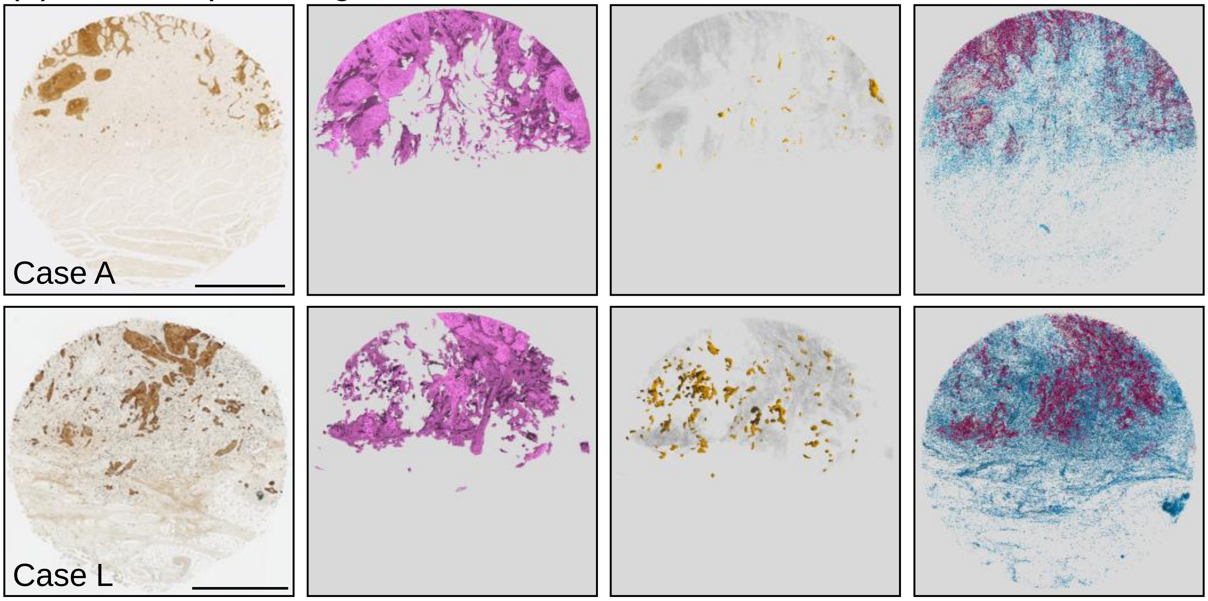

(4) Special forms

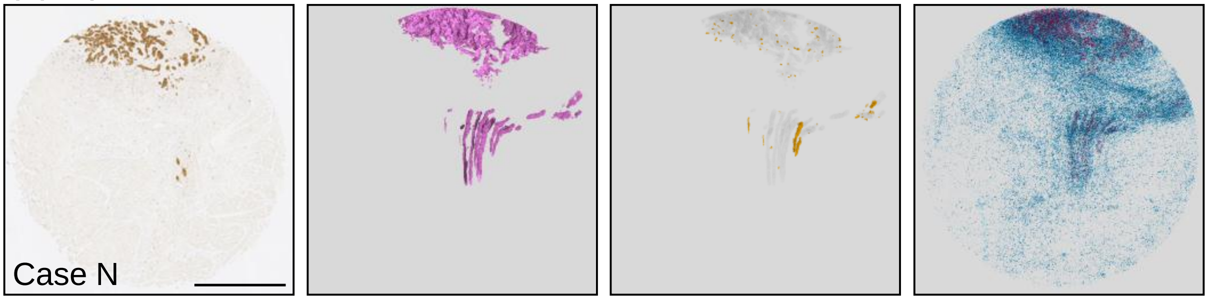

Supplement: Supplementary file 1 — Supplementary Material Plate S1: Microscopic images of pan-cytokeratin immunostained OTSCC lesions. A tissue core specimen of 3 mm in diameter was collected from the deep invasion front (indicated by the circle). The value indicates the depth of infiltration, except for case N where measurement was not applicable. Bar = 1 mm. [file 482765.f1.pdf]
